# Comparing Fly Ash Samples from Different Types of Incinerators for Their Potential as Storage Materials for Thermochemical Energy and CO_2_

**DOI:** 10.3390/ma12203358

**Published:** 2019-10-15

**Authors:** Saman Setoodeh Jahromy, Mudassar Azam, Florian Huber, Christian Jordan, Florian Wesenauer, Clemens Huber, Shaghayegh Naghdi, Karolina Schwendtner, Erich Neuwirth, Thomas Laminger, Dominik Eder, Andreas Werner, Michael Harasek, Franz Winter

**Affiliations:** 1 Institute of Chemical, Environmental and Bioscience Engineering, TU Wien, 1060 Vienna, Austria; mudassar.azam@tuwien.ac.at (M.A.); christian.jordan@tuwien.ac.at (C.J.); florian.wesenauer@tuwien.ac.at (F.W.); clemens.huber@tuwien.ac.at (C.H.); erich.neuwirth@tuwien.ac.at (E.N.); thomas.laminger@tuwien.ac.at (T.L.); michael.harasek@tuwien.ac.at (M.H.); franz.winter@tuwien.ac.at (F.W.); 2 Institute for Water Quality and Resource Management, TU Wien, 1040 Vienna, Austria; florian.huber@tuwien.ac.at; 3 Institute of Materials Chemistry, TU Wien, 1060 Vienna, Austria; shaghayegh.naghdi@tuwien.ac.at (S.N.); dominik.eder@tuwien.ac.at (D.E.); 4 Institute of Chemical Technologies and Analytics, TU Wien, 1060 Vienna, Austria; karolina.schwendtner@tuwien.ac.at; 5 Institute for Energy Systems and Thermodynamics, TU Wien, 1060 Vienna, Austria; andreas.werner@tuwien.ac.at

**Keywords:** municipal solid waste, fly ash, thermochemical energy storage, CO_2_ storage

## Abstract

This study aims to investigate the physical and chemical characterization of six fly ash samples obtained from different municipal solid waste incinerators (MSWIs), namely grate furnaces, rotary kiln, and fluidized bed reactor, to determine their potential for CO_2_ and thermochemical energy storage (TCES). Representative samples were characterized via simultaneous thermal analysis (STA) in different atmospheres, i.e., N_2_, air, H_2_O, CO_2_, and H_2_O/CO_2_, to identify fly ash samples that can meet the minimum requirements, i.e., charging, discharging, and cycling stability, for its consideration as TCES and CO_2_-storage materials and to determine their energy contents. Furthermore, other techniques, such as inductively coupled plasma optical emission spectroscopy, X-ray fluorescence (XRF) spectrometry, X-ray diffraction (XRD), scanning electron microscopy, leachability tests, specific surface area measurement based on the Brunauer–Emmett–Teller method, and particle-size distribution measurement, were performed. XRF analysis showed that calcium oxide is one of the main components in fly ash, which is a potentially suitable component for TCES systems. XRD results revealed information regarding the crystal structure and phases of various elements, including that of Ca. The STA measurements showed that the samples can store thermal heat with energy contents of 50–394 kJ/kg (charging step). For one fly ash sample obtained from a grate furnace, the release of the stored thermal heat under the selected experimental conditions (discharging step) was demonstrated. The cycling stability tests were conducted thrice, and they were successful for the selected sample. One fly ash sample could store CO_2_ with a storage capacity of 27 kg CO_2_/ton based on results obtained under the selected experimental conditions in STA. Samples from rotary kiln and fluidized bed were heated up to 1150 °C in an N_2_ atmosphere, resulting in complete melting of samples in crucibles; however, other samples obtained from grate furnaces formed compacted powders after undergoing the same thermal treatment in STA. Samples from different grate furnaces showed similarities in their chemical and physical characterization. The leachability test according to the standard (EN 12457-4 (2002)) using water in a ratio of 10 L/S and showed that the leachate of heavy metals is below the maximum permissible values for nonhazardous materials (except for Pb), excluding the fly ash sample obtained using fluidized bed technology. The leachate contents of Cd and Mn in the fly ash samples obtained from the rotary kiln were higher than those in other samples. Characterization performed herein helped in determining the suitable fly ash samples that can be considered as potential CO_2_-storage and TCES materials.

## 1. Introduction

Global population growth, massive urbanization, and economic growth have increased the consumption of natural raw materials and energy, resulting in huge amounts of waste. Since the resources are limited, sustainable resource management is necessary to secure the physical basis of society and economy in the long run [1]. Waste incineration is a key element in sustainable waste management, which decreases the amount and volume of solid waste and sanitizes the waste during incineration [2]. However, waste incinerators produce bottom ash and fly ash. The bottom ash can be deposited in nonhazardous-waste landfills or used as a road construction material.

Fly ash is particulate matter carried over from the combustion chamber and removed from the flue–gas stream prior to the addition of any type of sorbent material [3].

The composition and utilization of these fly ash samples depend on various factors, such as the fuel composition (feed), type of incinerator, air pollution control devices, and operating parameters. Therefore, these fly ash samples have diverse compositions.

In most cases, municipal solid waste is treated in grate furnace combustors [4], whereas sewage sludge and hazardous waste are mainly treated in fluidized bed combustors and rotary kilns, respectively [5,6,7,8]. Since current management practices for incinerating fly ash samples are associated with considerable environmental impacts [9], the utilization of these residues is desirable.

From this viewpoint, the possible applications for fly ash samples generated from municipal solid waste incinerators MSWIs have been the focus of many researchers during the last decade. For example, Ferreira et al. categorized the possible applications of fly ashes and discussed the advantages and disadvantages of their use in road construction, cement production, and pozzolanic materials [10]. Astrup et al. reported the management and utilization of fly ash in different European countries [11]. Generally, the application of municipal solid waste (MSW) fly ash samples are challenging, owing to their high chlorine concentrations, heavy metal content, and presence of persistent organic pollutants such as dioxin and furans, which increase the treatment and management costs.

Nonetheless, there are approaches that revalorize MSW fly ash and bottom ash in thermal energy storage, particularly in sensible heat storage, owing to the pozzolanic properties of ashes [12,13]. Cherbasnki et al. presented the CO_2_ sorption properties of fly ash for methane steam reforming [14]. Moreover, a possible application for MSWI fly ash samples as thermochemical energy storage (TCES) materials was reported by Setoodeh et al. [15]. TCES technology has been under development worldwide during the last decades, and interest in the industrial implementation of this type of energy storage system is increasing. In comparison with sensible and latent heat storage, TCES possesses advantages such as high energy density and unlimited energy storage duration without loss. However, TCES requires suitable and affordable materials for its operation to be comparable with other existing state-of-the-art storage technologies, such as sensible and heat energy storage. Investigations show that well-known TCES materials are suitable for different temperature levels [16,17,18,19,20]. Nonetheless, the screening of TCES materials has focused only on raw or doped materials. Overall, raw TCES materials possess drawbacks, such as sintering [21,22,23], slow conversion rates [22], low cycling stability [17], low thermal conductivity [17], and high costs [17,24], and researchers have attempted to overcome these drawbacks by doping or mixing raw TCES materials with other TCES/sensible heat storage materials [24,25,26,27,28]. For instance, the cycling stability of a calcium oxide (CaO)/CaCO_3_ system decreased with the number of cycles because the carbonation of CaO to form CaCO_3_ was significantly slow and sintering of material occurred at high temperatures [29]. Therefore, researchers investigated and improved the durability of the CaO/CaCO_3_ system by adding inert materials, such as TiO_2_ and LiSO_4_ [30,31]. Yanase et al. indicated that increasing the CeO_2_/CaO ratio led to a faster carbonation, and Erans et al. reported improved carbonation in the presence of water [30,32]. Jing et al. reported that adding Ca_3_Al_3_O_6_ can reduce the sintering effect of CaO/CaCO_3_ [33]. Chen et al. studied the impact of SiO_2_ doping on the CaO/CaCO_3_ system and reported an enhanced cycling stability, reduction of energy storage times, and higher released heat owing to the high thermal conductivity of SiO_2_ [26].

However, a material that can meet all the TCES requirements from the technical, environmental, and economic viewpoints remains under development. To the best of our knowledge, wastes or byproducts from industries have not yet been studied for use as TCES materials; however, the chemical compositions of MSW fly ash samples, their reduced or zero cost, harmful environmental impact, and amount produced per year make them an interesting material for further investigation regarding their revalorization as thermochemical and CO_2_ storage materials.

CaO, along with Al_2_O_3_, SiO_2_, and metal salts, is a main component of fly ash and a promising potential candidate for TCES systems [15]. One special feature of CaO is its ability to reversibly react with water vapor (H_2_O_(g)_) and carbon dioxide (CO_2_) to build two TCES systems (Equations (1) and (2)/at 25 °C).

Ca(OH)_2_ + ∆H_R_ ⇆ CaO + H_2_O   ∆H_R_ = 109 kJ/mol (1)

CaCO_3_ + ∆*H*_R_ ⇆ CaO + CO_2_   ∆*H*_R_ = 178 kJ/mol (2)

**Figure 1:**
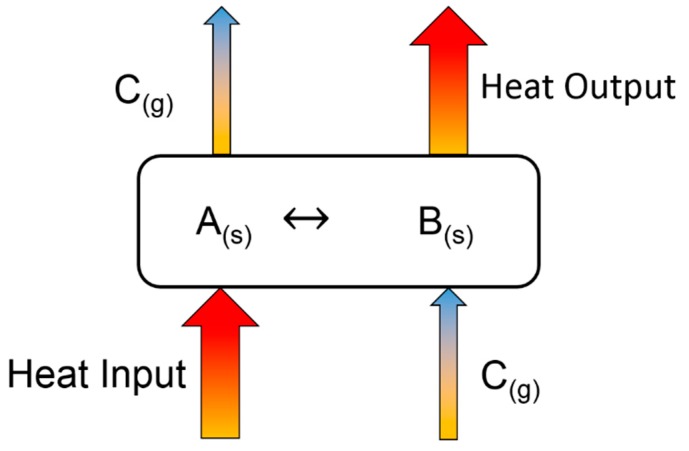
Principle of TCES system.

**Figure 2:**
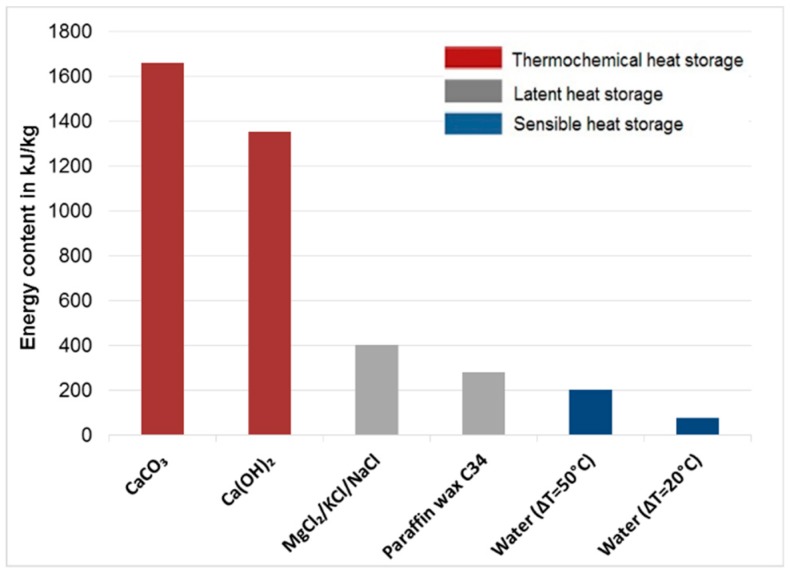
Comparison between energy contents of sensible, latent, and thermochemical energy storage technologies (carbon dioxide (CaCO_3_), calcium hydroxide (Ca(OH)_2_), molten chloride salt mixtures (MgCl_2_/KCl/NaCl)).

Figure 1 shows the basic principle of a TCES system [20]. Excess thermal heat from any plant, such as a concentrated solar power system, steam reforming plant, or MSWI, can be used as input heat to decompose a chemical component or mixture of different chemicals A_(s)_ (fly ash herein) into their components (B_(s)_ and C_(g)_) through endothermic reactions, known as the charging step (first requirement). The charged component B_(s)_ can be used for a period of time to produce component A_(s)_ on providing the required heat while reacting with gas component C_(g)_ through exothermic reactions, which is known as the discharging step (second requirement). The charging and discharging step involve heat consumption and release, respectively (Equations (1) and (2)). Figure 2 shows the comparison of the energy contents of different types of heat storage technologies compared with those of Ca(OH)_2_ and CaCO_3_ [34].

Based on Equation (2), fly ash can be carbonated by simultaneously harvesting energy. Hence, MSWI fly ash can be considered as a carbon capture and storage (CCS) material. The application of fly ash generated from coal-fired power plants (CFPPs) for CO_2_ capture and storage is discussed and reviewed earlier by Wee et al. and later by Dindi et al. [35,36]. Both papers concluded that fly ash from CFPPs can be considered as CCS materials in the raw or synthesized forms, such as zeolite or silica. Furthermore, they reported carbonation effect on the stabilization of fly ash for use as an admixture in cement (Dindi et al.) or for stabilizing harmful components in fly ash, such as Cd, Pd, As, and S [35,36].

Moreover, the carbonation of MSW fly ash and its positive impact on immobilizing heavy metals, such as lead and zinc, have been studied previously [37,38,39,40,41].

Harvesting and storing heat energy through reversible exothermic carbonation and endothermic decarbonation of fly ash is another advantage along with the aforementioned carbonation effect.

In our earlier investigation, three fly ash samples from the same technology (grate furnaces) were chemically characterized for their application as TCES materials. Results revealed that all three fly ash samples could store heat, and one fly ash could release the stored heat under designated conditions [15]. To obtain the complete spectra of various fly ash samples obtained from different types of incinerators to determine their TCES and CO_2_ storage potential, extensive characterization of samples from grate furnaces, rotary kiln, and fluidized bed was performed. 

**Figure 3:**
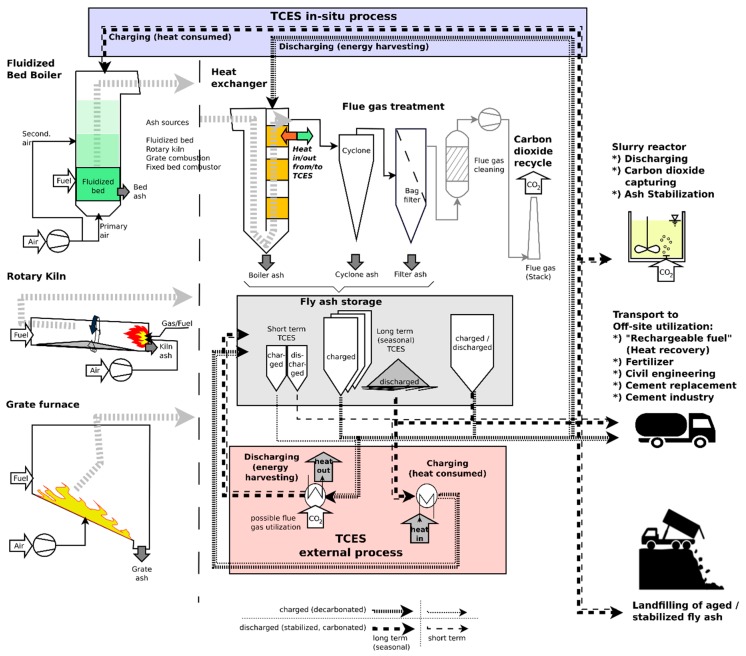
Schematic overview of process combination related to the utilization of fly ash as TCES or CO_2_ capture materials.

Different waste and biomass combustion technologies are shown in Figure 3 (left), which can provide fly ash for storage purposes. Fine fly ash particles can be separated from the flue–gas stream at the heat exchanger or at particle separators as boiler ash and cyclones or filters, respectively. Fly ash is stored in appropriate storage facilities according to the intended storage duration (short time or seasonal storage). Charging and discharging/CO_2_ binding of fly ash can be accomplished as an external process (using separate reactors) or by applying an in situ approach; discharging and CO_2_ fixation can be done by entering the charged (decarbonated) fly ash into the heat exchanger part of the plant and exposing it to CO_2_ in the flue gas. Charging is conducted by feeding the discharged (carbonated) fly ash into the burning chamber. As an alternative, discharging can also be conducted in a slurry reactor (achieving ash stabilization subsequently). It is also possible to utilize the energy storage material off-site by transporting it to remote energy extraction facilities by truck, train, or pipeline to make use of the stored thermal energy. Aged or excess fly ash can be used in cement industries and construction sites or as a stabilization material (binder) in landfill operations. Stabilized fly ash could also be landfilled directly. 

Owing to the existence of CaO in different phases, such as aluminate, silicate, and carbonate, all the fly ash samples were analyzed via X-ray diffraction (XRD). The determination of the particle-size distribution (PSD) and specific surface analysis based on the Brunauer–Emmett–Teller (BET) method were performed in addition to XRF, inductively coupled plasma optical emission spectroscopy (ICP-OES), and simultaneous thermal analysis (STA). STA measurements were performed to obtain information regarding the reactivity of fly ash samples in different atmospheres and to identify the fly ash samples that could meet the following criteria for a TCES material.

- Charging (endothermic reactions through thermal treatment)
  ○ A_(s)_ (fly ash/discharged) + heat ⇆ B_(s)_ (fly ash/charged) + C_(g)_
- Discharging (exothermic reaction by reacting with reactive gases, such as H_2_O and CO_2_)
  ○ B_(s)_ (fly ash/charged) + C_(g)_ ⇆ A_(s)_ (fly ash/discharged) + heat
- Cycling stability test (various charging and discharging of heat)

Furthermore, leachability test was performed to evaluate the environmental impact of fly ash. Scanning electron microscopy (SEM) analysis was conducted to determine the morphology changes before and after STA treatment up to 1150 °C in an N_2_ atmosphere.

## 2. Materials and Methods

### 2.1. Fly Ash Sampling and Waste Incineration Plants

Herein, six fly ash samples from five different types of waste incineration plants, i.e., rotary kiln, fluidized bed, and grate furnaces, were selected. Samples were collected at regular intervals over a period of four years. To obtain representative samples, the fly ash samples were combined and homogenized prior to their use for different analyses. As mentioned previously, fly ash characterization depends on the number of plant-linked parameters. Therefore, brief descriptions of the plants are given below.

Plant A

In plant A, MSW is treated in a grate furnace. Downstream from the boiler, activated coke is injected into the flue–gas stream to adsorb Hg, and this pollutant-loaded activated coke is separated together with fly ash from the flue gas using a filter separator. This filter ash is removed from the plant together with boiler ash. Furthermore, the dedusted flue gas is cleaned using a multi-stage scrubber and selective catalytic reduction (SCR) device.

Plant B

In plant B, MSW is treated in a grate furnace. The air pollution control and fly ash removal systems are identical to those in plant A.

Plant C

In plant C, MSW is treated in a grate furnace. Downstream from the boiler, ash is separated from the flue–gas stream using an electrostatic precipitator (ESP). In this plant, boiler ash and ESP ash can be removed and stored separately. Therefore, the two different ashes were sampled separately. Furthermore, the dedusted flue gas is cleaned in a multi-stage scrubber, SCR device, and fixed-bed activated coke filter.

Plant D

Plant D uses rotary kiln technology for treating hazardous waste. Downstream from the boiler, ash is separated from the flue–gas stream using an ESP. This filter ash is removed from the plant together with boiler ash. Then, the dedusted flue gas is cleaned using a multi-stage scrubber, SCR device, and fixed-bed activated coke filter.

Plant E

In plant E, refuse-derived fuel is treated in a fluidized bed combustor. Downstream of the boiler, ash is separated from the flue–gas stream using an ESP. This filter ash is removed from the plant together with boiler ash. Then, the dedusted flue gas is cleaned using a multi-stage scrubber, SCR device, and fixed-bed activated coke filter.

### 2.2. Chemical and Physical Analysis of Fly Ash Samples

Specific surface measurement was performed through physisorption (ASAP 2020, Micromeritics GmbH, Unterschleißheim, Germany). N_2_ gas was used, and the bath temperature used during the analysis was 195 °C. To eliminate the adsorbed gases and moisture from the sample, the sample was heated to 120 °C under vacuum overnight. The sample masses were 0.2–0.8 g.

PSD was measured using a laser diffraction analyzer (Mastersizer 2000, Malvern PANalytical, Almelo, Netherlands) and red and blue helium–neon light source at 633 and 466 nm, respectively. With this setup, it was possible to measure particle sizes in 50 different classifications of 0.02–2000 µm. All the samples analyzed herein were dry-dispersed at an injector air pressure of 2 bar. A sample mass between 2 and 10 g was placed on the vibrating gutter, and at least six measurements were performed for each sample to calculate an average value.

For the total content analysis of nonmatrix elements, fly ash samples were digested in aqua regia according to EN 13657 (2002) and subsequently analyzed according to EN 11885 (2009) using an ICP-OES (Optima 8300 ICP-OES spectrometer equipped with an SC-2 DX FAST sample preparation system) (Perkin Elmer, Waltham, MA, USA). A customized single-element standard was used for the calibration.

To determine the total content of matrix elements, samples were milled to a grain size of 250 µm and subsequently analyzed via X-ray fluorescence spectrometry (XRF, NITON XL3t Air) (Thermo Fischer Scientific, Waltham, MA, USA).

Powder XRD measurements were performed on a PANalytical X’pert-Pro diffractometer (CuKα, 45 kV, 40 mA, continuous scan, Soller slits 0.04 rad, Bragg–Brentano HD mirror, X’Celerator detector, 2Θ range 5°–70°, 200 s/step measurement time) (PANalytical, Almelo, Netherlands). The representative samples were manually ground in an agate mortar for 5–10 min and mounted on a zero-background sample holder with minute amounts of grease. Evaluation and phase identification were performed using the search and match routine of the PANalytical HighScore Plus Program Suite on the ICDD database (ICDD, 2017) [42]. This was followed by Rietveld refinement with TOPAS software (Brucker, Billerica, MA, USA) using CIF files from the ICSD database [43,44].

The STA data obtained in the different atmospheres were generated on a Netzsch STA 449 Jupiter instrument equipped with a TGA–DSC sample holder (Erich Netzsch GmbH & Co. Holding KG, Selb, Germany). This instrument maintained a water-vapor furnace, including an air-cooled double jacket, heated vapor inlet, heated transfer line, and heated collar. The oven had the capacity to operate between 25 °C and 1250 °C, regulated by an S-type thermocouple. All samples were measured in aluminum oxide crucibles using various gas atmospheres (N_2_, N_2_/O_2_ (air), CO_2_, H_2_O, and CO_2_/H_2_O). Some samples, which demonstrated melting or were not separable from the crucible after decomposition (fly ash samples D and E), were not tested in all conditions (see Table 1). The oven was heated at 30 °C/min to 1240 °C, setting a sample temperature of approximately 1150 °C for all experiments. Table 1 lists an overview of the performed experiments.

A cycling stability test was performed for the selected fly ash sample. The sample (30 mg) was heated to 880 °C at a heating rate of 30 °C/min in an N_2_ atmosphere (charging step). After a stabilization time of 30 min, the atmosphere was changed to a mixture of CO_2_ (100 ml/min) and water vapor (1 g/h), cooled at 10 °C/min from 880 °C to 350 °C and maintained at 350 °C for 30 min. This cycle was repeated thrice.

A FEI Quanta 250 FEGSEM microscope (FEI, Hillsboro, OR, USA) was used for SEM images. These were recorded at a 5-kV acceleration voltage under a high vacuum of 6 × 10^−6^ mbar. A magnification of 1000 shows the thermal treatment of samples very well compared with higher resolutions. In accordance with the standard EN 12457-4 (2002), the leachability test was performed on the original samples. 

## 3. Results and Discussion

### 3.1. BET Surface Area

The BET surface areas are listed in Table 2. Fly ash samples A and B (grate furnaces) display the highest surface areas of 3.6 and 4.3 m^2^/g, respectively, followed by fly ash C (filter ash from grate furnace) with a surface area of 2.1 m^2^/g. Other fly ash samples have average surface areas of 1.6 ± 0.1 m^2^/g. Fly ash samples A and B have higher BET surface areas owing to the higher unburned carbon contents or activated carbon added to the fly ash sample in the air pollution control system. The lower BET surface areas of fly ash samples correspond to the finest fractions, which contain less unburned carbon and higher inorganic content [45].

### 3.2. PSD Analysis

**Figure 4:**
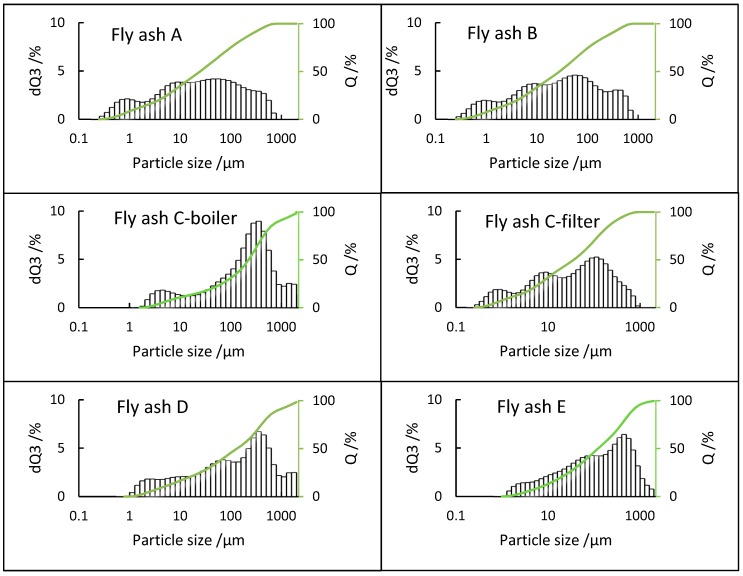
PSDs of all fly ash samples, dQ3 (volume %), and Q (cumulative distribution %).

PSD analysis of fly ash samples plays a vital role in assessing and evaluating their potential utilization and environmental impact since it directly influences their characteristics [46]. A comparison of PSD results is shown in Figure 4, which indicates that only boiler ash sample C-boiler has a bimodal distribution with modes at 4 and 315 µm and large fraction of coarse particles reaching 2000 µm. Fly ash sample C-filter has a multimodal distribution with modes at every magnitude from 1 to 100 µm. It is noteworthy that there are no particles larger than 1000 µm. Samples A and B are from different plants having the same combustion process and parameters. Both show a bimodal distribution of 0.2–1000 µm with modes at approximately 1, 10, and 100 µm. Fly ash sample E shows unimodal distribution in the range 1–2000 µm with mode at 400 µm. Fly ash sample D also shows a multimodal distribution in the range 1–2000 µm with notable mode at 315 µm. Regarding the E, D, and C boilers, coarse particles reach 2000 µm. It can be interesting to investigate their elemental compositions to identify the size fractions, wherein elements such as Ca accumulate; the same is applicable to the fine grain size fraction of approximately 1 µm of the A, B, and C-filters.

### 3.3. Chemical Composition of Fly Ash Samples

The total content of matrix elements determined by XRF is listed in Table 3, and the total content of nonmatrix elements determined by ICP-OES is listed in Table 4. Regarding the XRF results, the elemental content is calculated for the oxide form. Most elements should exist in their oxide form; however, some are present as carbonates, sulfates, and possibly chlorides. Investigated fly ash samples mainly comprise Na_2_O, SiO_2_, Al_2_O_3_, SO_3_, Cl, and CaO. The boiler ash from plant C shows the highest content of CaO, which appears to be the most suitable compound for TCES investigations. The remaining fly ash samples have similar CaO-content ranges, except for fly ash sample D, which contains less CaO than all the other samples. Fly ash samples D and E have the highest SiO_2_ and sulfur contents, which, in fly ash sample E, is due to the high velocity in the fluidized bed reactor, partially removing the bed material (sand), a major source of SiO_2_. Plants A, B, C-filter, and E produce fly ash with similar CaO contents.

Furthermore, based on the ICP-OES analysis, plant C-boiler ash shows low values of potentially hazardous elements, such as Pb, Cd, Hg, and As, in comparison with other fly ash samples from grate furnaces (A, B, C-filter). Fly ash samples A, B, and C-filter show similarities in their matrix and nonmatrix elements. Fly ash sample D shows a higher Pb content than the other fly ash samples.

All fly ash samples contain high zinc contents, exceeding 18000 mg/kg, except those from plants C-boiler and E. Zn can be recovered using the FLUWA/FLUREC process, which has already been fully applied in Switzerland [47,48,49]. Similar research was conducted by Fellner et al. on zinc (Zn) recovery from fly ash samples in Europe [4].

### 3.4. XRD Analysis

**Figure 5:**
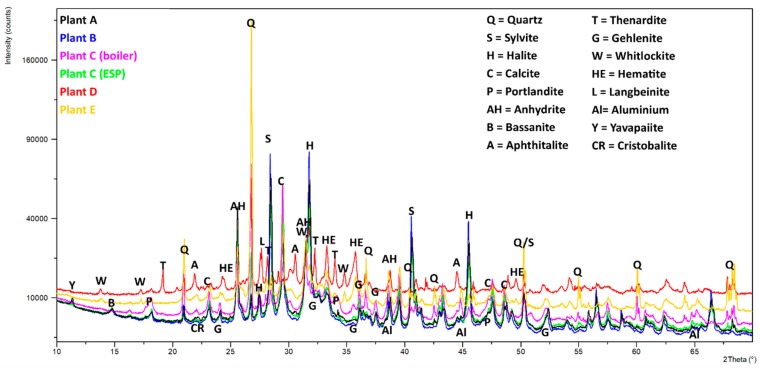
Powder XRD graphs (Cu Kα) of the six samples. The peaks of the main constituents (see Table 5) are included. A visible difference in the background height is due to sample fluorescence (Fe) and possible differences in the amorphous content.

**Figure 6:**
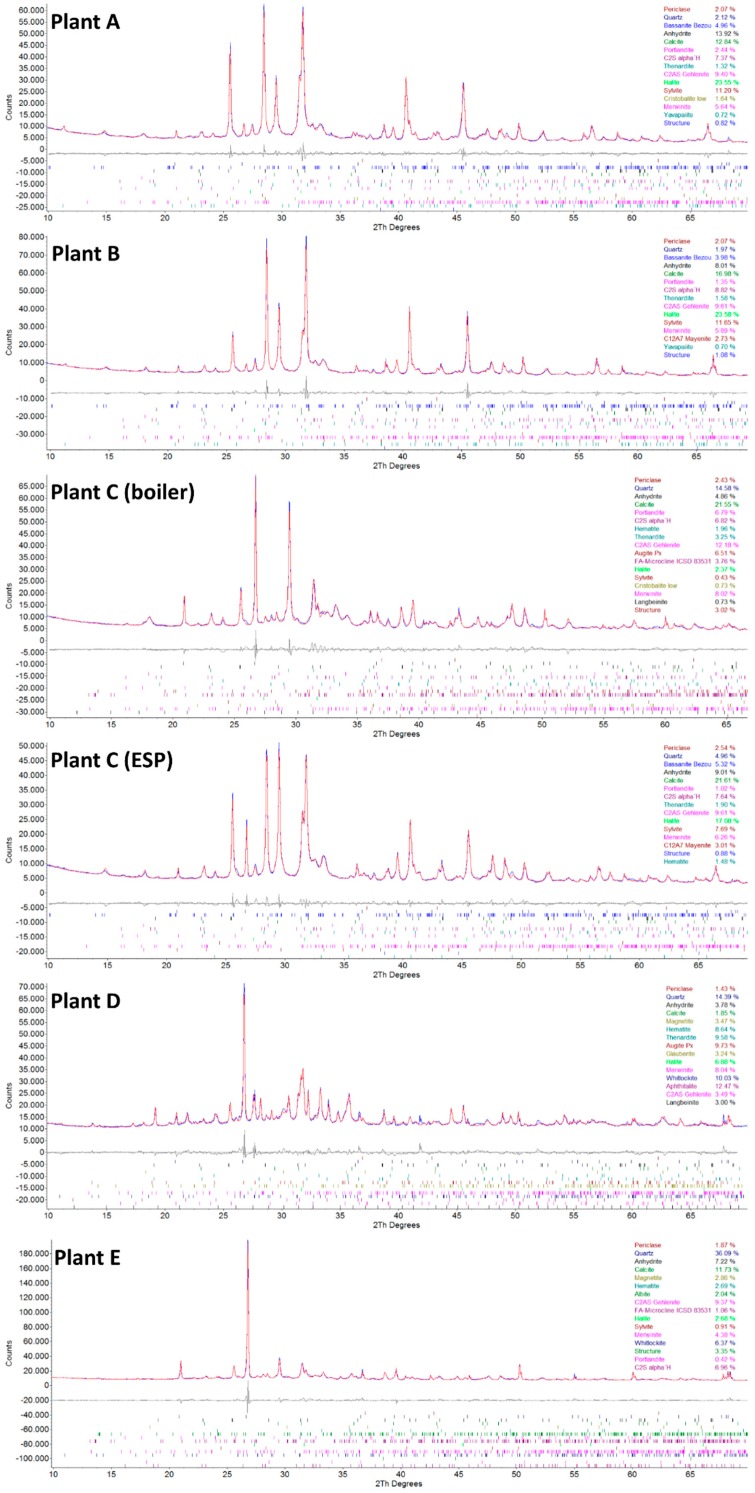
Powder XRD graphs showing the results of the Rietveld refinements (blue = measured diffractogram, red = calculated diffractogram, and gray = difference plot) of plant **A** (fly ash sample A), plant **B** (fly ash sample B), plant **C** (boiler) (fly ash sample C-boiler), plant **C** (ESP) (fly ash sample C-filter), plant **D** (fly ash sample D), and plant **E** (fly ash sample E). The calculated phase percentage is given, and the peak positions are calculated. The possible amorphous contents were not evaluated. The difference curve shows a good fit for the main peaks; however, minor phases that could not be clearly identified probably exist.

Qualitative phase analysis shows that all samples contained extremely complex phase compositions (Figure 5 and Figure 6). The peak search routines were only helpful for the 4–6 main compounds, but Rietveld refinements revealed that various additional phases must be present. The six compounds mostly comprised different amounts of quartz, anhydrite, calcite, halite, and sylvite. Aluminosilicates were present in the form of belite, mayenite, and gehlenite; however, feldspars and pyroxenes were also found in some samples. If phosphate is present in larger amounts, it gets crystallized as whitlockite. Most samples contained metallic aluminum in low amounts, and iron was found in the form of hematite (see Table 5).

Samples C-filter, A, and B had very similar phase contents, containing large amounts of salts (NaCl, KCl, and CaSO_4_). Sample D mainly contained salts in the form of sulfates (Na_2_SO_4_ and K_3_Na(SO_4_)_2_).

Most samples contain portlandite (Ca(OH)_2_), which is probably an alteration product of free lime (CaO), which reacts very quickly with moisture in the air.

### 3.5. Thermogravimetric Analysis and Differential Scanning Calorimetry (TGA/DSC) of Fly Ash Samples

The fly ash samples from grate furnaces, rotary kiln, and fluidized bed combustors were analyzed by STA in different atmospheres, such as N_2_, synthetic air, CO_2_, H_2_O, and H_2_O/CO_2_.

**Figure 7:**
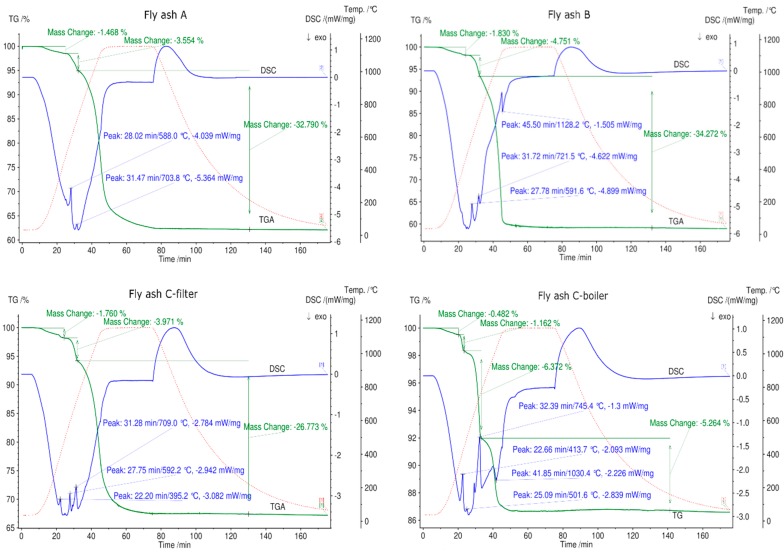

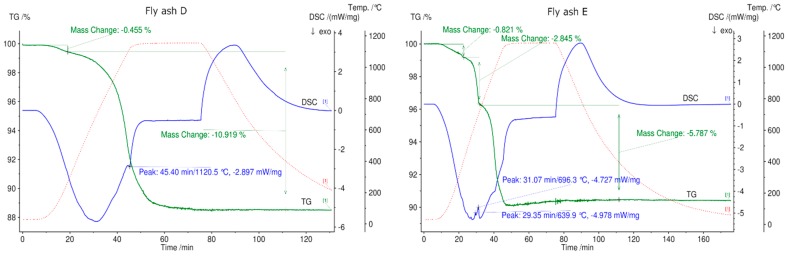
STA results for all fly ash samples in a N_2_ atmosphere between 30 °C and 1150 °C. The green line denotes percentage mass loss, blue line denotes DSC trace, and dotted red line denotes temperature profile.

**Figure 8:**
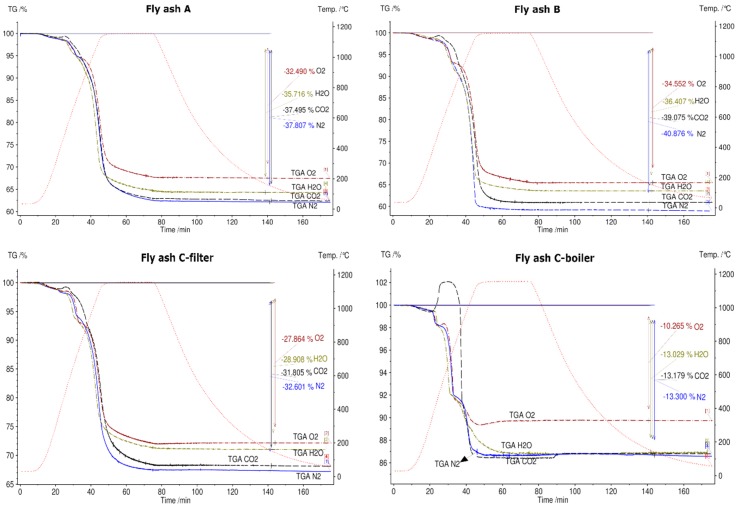
Thermogravimetric (TG) results for fly ash samples **A**, **B**, **C**-filter, and **C**-boiler in different atmospheres, namely N_2_, synthetic air, H_2_O, and CO_2_, between 30 °C and 1150 °C.

**Figure 9:**
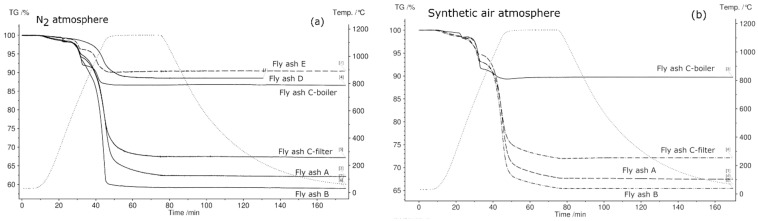

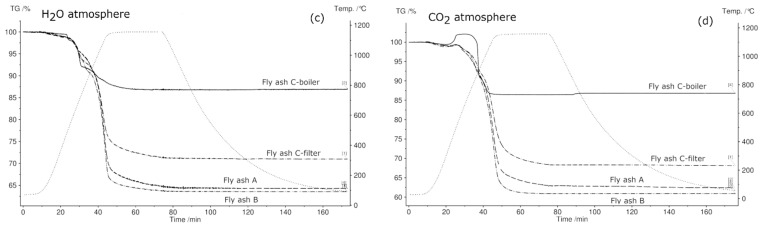
Comparison of thermogravimetric (TG) curves of fly ash samples in N_2_ (**a**), synthetic air (**b**), H_2_O (**c**), and CO_2_ (**d**) in the temperature range 30 °C–1150 °C.

The STA results are given in Figure 7, Figure 8 and Figure 9. Fly ash samples A, B, and C-filter from grate furnaces demonstrate three similar steps of mass change (mass loss) within the investigated temperature range (30 °C–1150 °C). The mass loss was constant at 1150 °C for 30 min at this temperature.

The first mass loss up to 500 °C occurs because of desorption of physically and chemically adsorbed water combined with volatilization of organic components and volatile elements existing in fly ash samples [15,50,51]. The mass loss continues in the temperature range 530 °C–770 °C, and an average mass loss of approximately 4% is observed for fly ashes A, B, and C-filter. This step demonstrates the decomposition of Ca(OH)_2_ or other metal hydroxides existing in fly ash samples [15]. The last step of 770 °C–1150 °C indicates the breakdown of carbonate, sulfate, aluminum salts, and chlorides [15,50,51]. The DSC signals confirm endothermic reactions at the second and third steps of the mass loss for fly ash samples A, B, and C-filter, which are related to the dehydration of hydroxides and decomposition of carbonates and sulfates, respectively. C-filter differs in mass loss in the third step compared to fly ashes A and B due to differences in the sulfate, carbonate, and chloride contents.

Fly ash sample C-boiler shows four steps of mass loss. The total mass change of fly ash sample C-boiler is significantly less than those of A, B, and C-filter. XRF analysis indicated high SiO_2_ and Al_2_O_3_ contents in fly ash sample C-boiler in comparison with the other fly ash samples. These compounds contribute less toward the mass loss with respect to the inert behaviors of these components.

Fly ash sample D from a rotary kiln shows an almost continuous mass loss. A small step with 0.5% mass loss up to 300 °C can be observed, which is related to the desorption of physically and chemically adsorbed water. This type of fly ash contains more sulfates and sodium, such as Na_2_SO_4_ and K_3_Na(SO_4_)_2_, confirmed by XRD, and less Ca, as confirmed by XRF analysis. A mass loss of 11.3% occurs over the entire temperature range for this type of fly ash. Fly ash sample E shows three steps of mass loss over the total temperature range up to 1150 °C with the least mass loss of below 10% in total. This can be attributed to fly ash sample E having the highest silicon oxide content based on XRF analysis.

The relative mass changes with the appropriate temperature ranges and reaction types are listed in Table 6.

The different mass loss trends can be explained based on the variations in feed, combustion technology, bed temperature, and gas cleaning system.

After conducting the first TGA experiment on all fly ash samples in a N_2_ atmosphere, it was observed that samples from rotary kiln D and fluidized bed E were melted and they were not removable from the crucibles.

Figure 8 shows the TGA curves of fly ashes from plants A, B, C-filter, and C-boiler in different atmospheres, namely N_2_, air, H_2_O, and CO_2_. Fly ash samples A, B, and C-filter show the same trend of curves in different atmospheres, whereas fly ash C-boiler shows a different trend. The TGA signal of fly ash C-boiler in a CO_2_ atmosphere exhibits different behavior. It is noteworthy that at 410 °C, fly ash samples A, B, and C-filter displayed mass losses, whereas C-boiler ash showed a 2.7% (*w*/*w*) mass increase. This direct carbonation with L/S = 0 shows a CO_2_-storage capacity of 27 kg CO_2_/ton fly ash. CO_2_ sequestration of coal power plants varies in the range between 265 kg CO_2_ to 25.2 kg/ton fly ash depending on the chemical composition and carbonation procedures performed, i.e., direct or indirect [36]. 

The trend of reduced mass loss was observed during all the experiments that were conducted in synthetic air. The total mass loss is least in the synthetic air atmosphere due to oxidation of metals, which reduces the mass change. The maximum mass changes in all fly ash samples were detected in the N_2_ atmosphere. The total mass loss for fly ashes A, B, and C-filter in water vapor and CO_2_ atmospheres showed intermediate behavior with respect to the total mass loss in comparison to those in N_2_ and O_2_ atmospheres. The total mass loss was constant for fly ash C-boiler in CO_2_ and water vapor and N_2_ atmospheres.

Figure 9 shows the thermogravimetric (TG) signals of the experimental samples in N_2_, synthetic air, H_2_O, and CO_2_ atmospheres. In N_2_ atmosphere, the fly ash sample generated from the grate furnace in plant B showed the maximum mass loss and that from fluidized bed E showed the minimum mass loss. As mentioned previously, fly ash samples D and E were not subjected to STA in atmospheres other than N_2_ because they exhibited a phase change during heating in N_2_ atmosphere, questioning their suitability as TCES materials. In synthetic air, CO_2_, and H_2_O atmospheres, fly ash sample B exhibited the highest mass loss and fly ash sample C-boiler exhibited the lowest mass loss. The percentage mass changes in fly ash samples in different atmospheres are shown in Figure 9.

**Figure 10:**
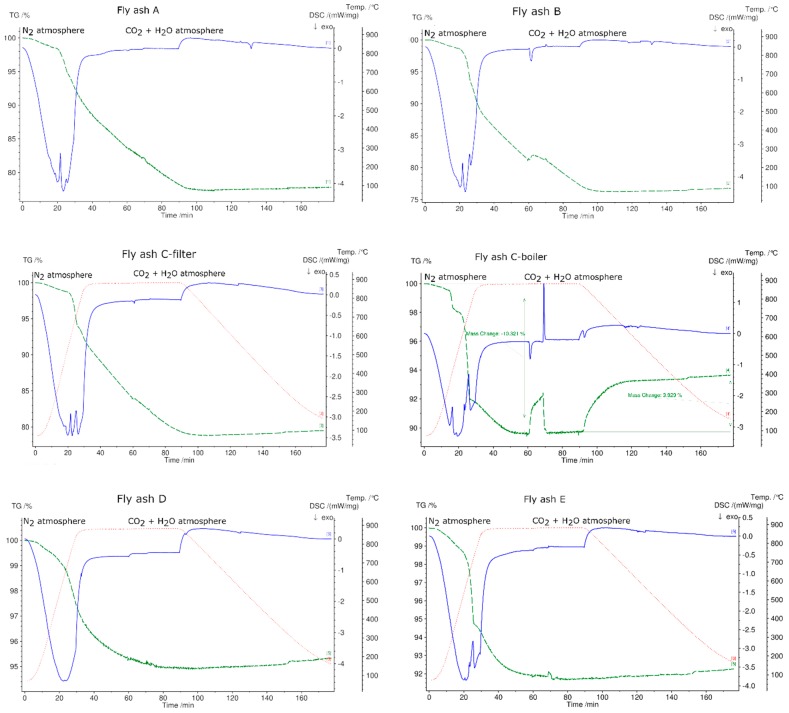
Charging and discharging of fly ash samples in STA. Charging at a heating rate of 30 °C/min to 880 °C in N_2_ atmosphere and discharging of fly ash samples in pure CO_2_ (100 ml/min) with added water vapor H_2_O (1 g/h) at a cooling rate of 10 °C/min. Green line denotes the percentage of mass loss, blue line denotes DSC signal, and dotted red line denotes temperature profile.

Figure 10 shows the charging and discharging steps for all fly ash samples. The charging steps were performed in N_2_ atmosphere up to 880 °C, whereas discharging was performed in CO_2_ and H_2_O atmosphere, and samples were cooled to 70 °C at a rate of 10 °C/min. All fly ash samples underwent exothermic reactions followed by mass loss, despite fly ash sample D showing no endothermic peaks in its DSC signal. Only fly ash sample C-boiler showed the reverse reaction under these experimental conditions. Up to 880 °C, the fly ash can store 226 kJ/kg (integrations of DSC peaks with linear base line) followed by a mass loss of 10.3%. The thermal energy released during the reverse reaction was 86 kJ/kg based on 38% conversion according to the mass signal.

### 3.6. Energy Density

To determine the energy content of fly ash samples on thermal treatment up to 1150 °C, the endothermic peaks generated in DSC signals were integrated with a linear base line; results are listed in Table 7.

Fly ash sample D shows no endothermic peak, whereas fly ash sample C shows the highest energy content of 394 kJ/kg.

The measured energy content of fly ash C-boiler (394 kJ/kg) is not negligible in comparison with potential metal oxide candidates at high temperatures, such as Co_3_O_4_/CoO, CuO/Cu_2_O, Mn_2_O_3_/Mn_3_O_4_, and MnO_2_/Mn_2_O_3_ with energy content values of 844, 810, 202, and 480 kJ/kg, respectively, in TCES [27,28,34].

### 3.7. Cycling Stability Test

**Figure 11:**
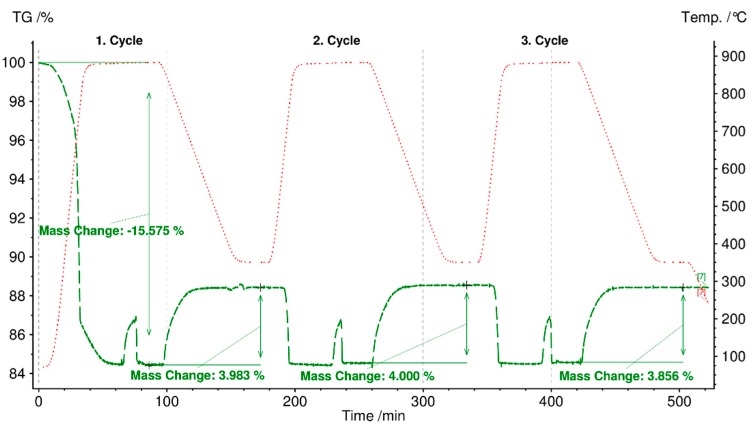
Cycling stability test of fly ash C-boiler heated to 800 °C at a heating rate of 30 °C/min in a N_2_ atmosphere (charging step) and cooled from 800 °C to 350 °C at a cooling rate of 10 °C/min in CO_2_ (100 ml/min) and H_2_O (1 g/h) atmosphere.

Figure 11 shows cycling stability test results for three cycles of selected fly ash C-boiler because this meets the first (charging) and second requirements (discharging).

The initial mass loss of 15% up to 880 °C in a N_2_ atmosphere (charging) is due to the decomposition of organic compounds, hydroxides, carbonates, and sulfates, and vaporization of light heavy metals, such as Zn, Cd, and their chloride compounds [15,51,52]. The reverse reaction occurs through the carbonate of fly ash with a mass gain of 3.9%, starting at 100 min for the first cycle. The second discharging cycle continued at 200 min with a mass loss of 4% in N_2_ atmosphere, and the reverse carbonation reaction was completed at 300 min with an expected mass increase of 4%. The same trend of mass changes was observed for the third discharging and charging steps. Hence, based on the experiment results, the third requirement for TCES material, i.e., cycling stability (charging and discharging), can be met by fly ash C-boiler for three cycles. The carbonation reaction time under the designated experimental conditions was set at 85 min for each cycle (cooling from 880 °C to 350 °C and maintaining at 35 °C for 30 min). Results showed that the reaction for each step was completed faster, i.e., in 40 min, than the set time of 85 min.

Table 8 lists the specific energy contents of decomposition (charging) up to 880 °C and reverse reaction of the decomposed form of fly ash C-boiler with gas components (100 ml/min CO_2_ and 1 g/h H_2_O). The measured energy content for charging is based on integration of DSC peaks with a linear baseline and that for discharging is obtained from the mass signal.

### 3.8. Scanning Electron Microscopy

**Figure 12:**
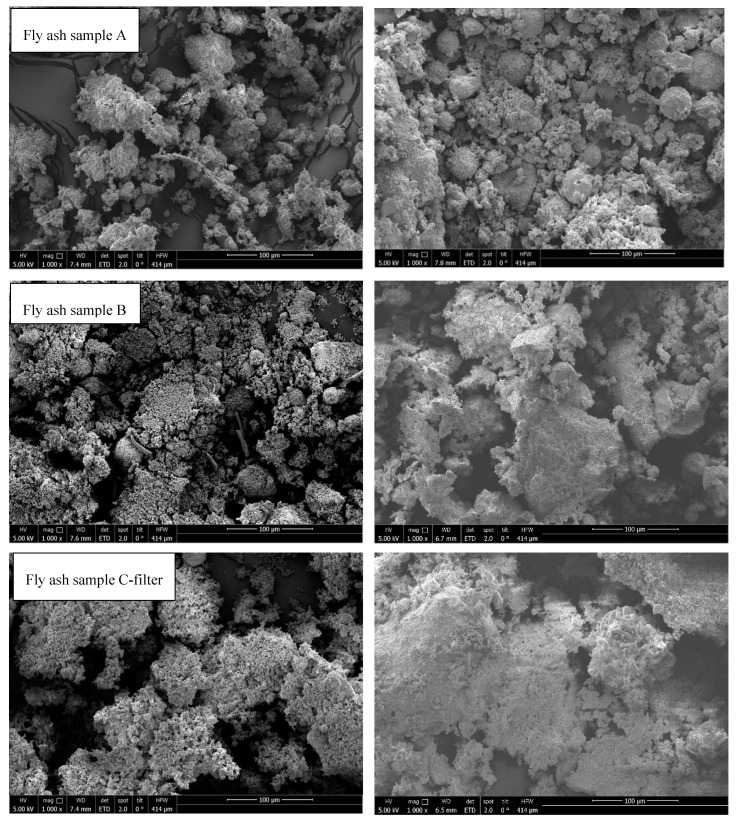

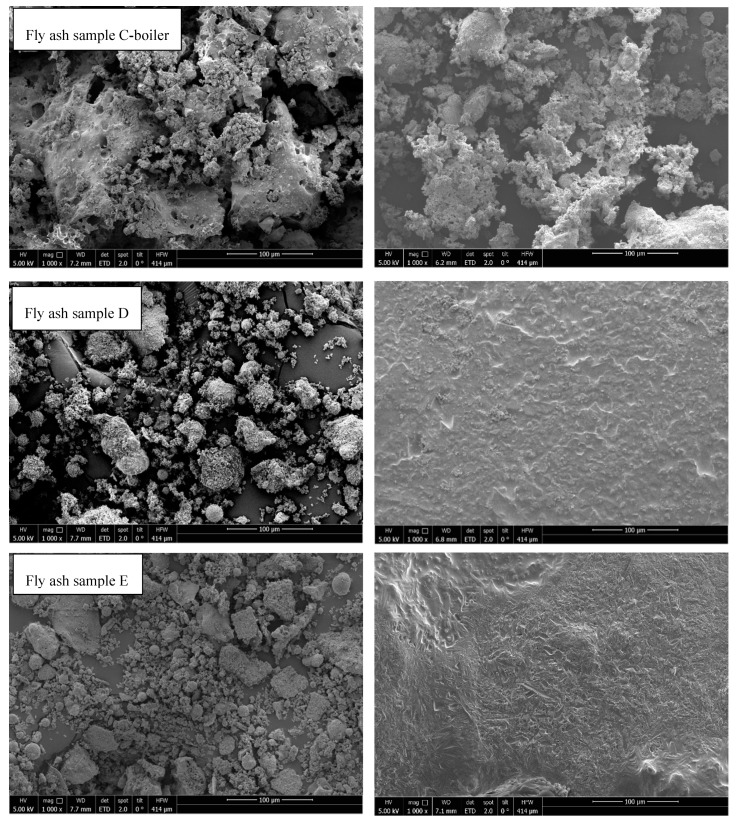
SEM analysis of fly ash samples before (left) and after (right) STA experiments.

SEM analysis was performed to determine the changes in the particle morphology after STA treatment. Figure 12 shows the thermal effects on all samples. Fly ash samples from rotary kiln (fly ash sample D) and fluidized bed (fly ash sample E) were melted. In all the other samples, agglomeration and sintering were observed; however, samples were in compact powder form and could be removed from the crucible. On the contrary, fly ash C-boiler was less affected by thermal treatment from an optical viewpoint. Agglomeration and sintering effects can negatively impact the cycling stability of a material in TCES, which should be considered in further investigations.

### 3.9. Leaching Test

Ashes from combustors are exposed to different atmospheres and natural processes throughout storage, utilization, or disposal, which may trigger contamination of water sources by the leaching of heavy metals [53]. Different leaching tests have been conducted to determine the affinity of these metals with their surroundings. The leaching capacity of these metals is pH dependent; therefore, pH is the dominant factor in defining their leaching rate [54]. Leaching analysis plays a vital role in assessing the utilization and treatment of fly ash samples [3,53].

The leaching test results (EN 12457-4) are presented in Table 9. The limit values are the lower limits for non-hazardous-waste landfill, whereas the highest limits are listed in brackets, as set by the Austrian landfill ordinance [15,55]. The Pb leaching values of samples A, B, and C-filter are considerably higher than in the nonhazardous-waste category. The Mn and Cd leaching contents from fly ash sample D from a rotary kiln were significantly higher than the limit values or values from other fly ash samples. The leaching contents of other elements and components were all below the limit values.

## 4. Conclusions

Herein, a detailed chemical and physical characterization of fly ash samples obtained from MSWI, such as grate furnaces, fluidized beds, and rotary kilns, was performed to determine the possible utilization of fly ash as TCES and CO_2_-storage materials. The main conclusions from the performed analyses are summarized as follows:

- Based on the SSA results obtained between 1.5 and 4.3 m^2^/g, no specific relation between their values and potential usability of fly ashes for TCES and CO_2_ storage was found. To determine the sorption potential, further analysis on the pore volume, pore size distribution, average pore diameter, and narrow microporosity parameters should be performed.
- The PSDs of fly ash samples E (fluidized bed), C-boiler (grate furnace), and D (rotary kiln), showed unimodal, bimodal, and multimodal distributions, respectively. Fly ashes A and B from grate furnace technologies with the same combustion processes showed the same PSDs with modes at appropriately 1, 10, and 100 µm.
- The fly ash sample C-boiler differs in Ca amount compared to other fly ash samples via XRF analysis. According to XRD results, no free CaO was detectable. However, portlandite Ca(OH)_2_ and calcite (CaCO_3_), which are most likely the alteration products of free CaO, were present in most samples. Alteration and its effect on free CaO content and subsequently on its potential for TCES and CO_2_ capture should be investigated separately.
- Based on TGA results up to 1150 °C in N_2_ atmosphere, fly ashes A, B, C-filter, C-boiler (grate furnace), D (rotary kiln), and E (fluidized bed) achieved total mass losses of 37%, 40%, 32%, 13%, 11%, and 9%, respectively. Fly ash sample C-boiler had the highest energy content of 394 kJ/kg, and no endothermic peaks were detected for fly ash sample D. Fly ash samples A, B, C-filter, and E possessed energy contents of 94, 85, 98, and 50 kJ/kg, respectively. This showed that all fly ash samples, except fly ash sample D from a rotary kiln, met the first requirement of TCES under the selected experimental conditions, which is to store thermal heat through endothermic reactions.
- The second requirement for a TCES material (exothermic reaction by reaction with reactive gas components (H_2_O and CO_2_)) was met by fly ash sample C-boiler. The energy content in fly ash sample C-boiler obtained by thermal treatment up to 880 °C was approximately 226 kJ/kg, approximately 86 kJ/kg of which could be released under the selected operational conditions in STA.
- Cycling stability tests, i.e., various charging and discharging of heat, as the third requirement for TCES materials, were accomplished for fly ash sample C-boiler for three cycles in STA. The charging step occurred at a heating rate of 30 °C/min up to 880 °C for 30 min, followed by heat storage between 99 and 290 kJ/kg. Discharging started at a cooling rate of 10 °C/min from 880 °C to 350 °C, maintaining at 350 °C for 30 min in CO_2_ and H_2_O vapor atmospheres, and followed by heat release between 73 and 101 kJ/kg.
- Fly ash sample C-boiler could store CO_2_ with a storage capacity of 27 kg CO_2_/ton fly ash.
- According to ICP-OES analysis and leachability test, the fly ash C-boiler showed that this kind of fly ash may be considered to be nonhazardous, making this fly ash more feasible since it does not exceed the limits for disposal in standard above-ground landfills. However, before it can be considered to be a nonhazardous material such as total organic carbon, further analysis of its total dissolved solids needs to be performed.
- SEM analysis of all samples before and after thermal treatment by STA up to 1150 °C in N_2_ atmosphere revealed sintering, agglomeration, and melting of particles. However, fly ash samples were in compact powder form and easily removable from the crucible, except for fly ashes D (rotary kiln) and E (fluidized bed) because they melted.

It can be concluded that among all fly ash samples analyzed herein, fly ash sample C-boiler could meet the minimum requirements, i.e., charging, discharging, and cycling stability, for consideration as TCES material and can also be considered as CO_2_ storage material based on direct and dry carbonation results in a TGA experiment. This study aims to bring awareness among researchers in the field of TCES to focus on raw or doping materials for producing an optimal TCES material and to take into account the byproducts and waste from industries as well. Furthermore, this study can contribute toward developing the practical application of an environmentally-sustainable solid waste management strategy. However, due to issues such as legislation, which varies from country to country, reasonable system integration considering all points regarding the potential uses of TCES, including CCS, stabilization of fly ash through carbonation by simultaneously harvesting energy, economic aspects, and life cycle assessment, must be separately investigated.

## Figures and Tables

**Table 1:** An overview of the STA experimental runs for different fly ash samples ((x) for performed and (–) for not performed).

Atmosphere	Fly Ash Sample A	Fly Ash Sample B	Fly Ash Sample C-filter	Fly Ash Sample C-boiler	Fly Ash Sample D	Fly Ash Sample E
N_2_	x	x	x	x	x	x
O_2_	x	x	x	x	–	–
CO_2_	x	x	x	x	x	x
H_2_O	x	x	x	x	–	–
H_2_O/CO_2_	x	x	x	x	x	x

**Table 2:** BET surface areas of fly ash samples in m^2^/g.

Fly Ash Samples	A	B	C-boiler	C-filter	D	E
BET surface area	3.69	4.36	1.61	2.15	1.79	1.56

**Table 3:** Total content of matrix elements in different fly ash samples determined by XRF.

Elements	Fly Ash A	Fly Ash B	Fly Ash C-boiler	Fly Ash C-filter	Plant D	Plant E
Na_2_O	+++	+++	+	+++	+++	+
MgO	+	+	+	+	+	+
Al_2_O_3_	++	++	+++	++	+	+++
SiO_2_	+++	+++	++++	+++	+++	+++++
P_2_O_5_	+	+	+	+	+	+
SO_3_	++	++	++	++	+++	+
Cl	+++	+++	+	+++	+	+
K_2_O	++	++	+	++	+	+
CaO	++++	++++	+++++	++++	+++	++++
TiO_2_	+	+	+	+	+	+
Fe_2_O_3_	+	+	+	+	++	+

+++++, ++++, +++, ++, and + represent very high, high, medium, low, and very low intensities, respectively.

**Table 4:** Total content of nonmatrix elements in different fly ash samples determined by ICP-OES given in mg/kg, where LOQ = limit of quantification.

Elements	Plant A	Plant B	Plant C-boiler	Plant C-filter	Plant D	Plant E
Sb	742	635	302	1316	656	127
As	37	32	13	37	83	7
Ba	927	984	1745	1048	183	809
Pb	4883	4394	1100	7016	12948	1254
Cd	347	321	38	375	321	14
Cr	245	259	276	242	780	234
Co	24	27	38	35	111	27
Cu	1008	1004	1009	1901	3315	3608
Mn	574	676	1050	816	816	676
Mo	24	20	22	24	191	10
Ni	59	60	152	95	615	109
Hg	12.50	34.79	0.34	4.34	0.21	<LOQ
Ag	49	44	14	47	126	12
Zn	20722	18371	5684	21613	38108	3870
Sn	948	835	177	865	1154	147

**Table 5:** XRD-Rietveld refinement results of six compounds (given in wt % and normalized to 100%). Amorphous contents and unidentified minor components are probably present but could not be accounted for. Therefore, the quantification error is estimated at 20% relative and minimum of 2% absolute.

Compound	Chemical Formula	A	B	C-Filter	C-Boiler	D	E
Aluminum	Al	1	1	1	3	3	3
Quartz	SiO_2_	2	2	5	15	14	36
Cristobalite	SiO_2_	2			1		
Hematite	Fe_2_O_3_			1	2	9	3
Magnetite	Fe_3_O_4_					3	3
Portlandite	Ca(OH)_2_	2	1	1	7		0
Periclase	MgO	2	2	3	2	1	2
Calcite	CaCO_3_	13	17	22	22	2	12
Anhydrite	CaSO_4_	14	8	9	5	4	7
Bassanite	CaSO_4_x0.5H_2_O	5	4	5			
Aphthitalite	K_3_Na(SO_4_)_2_					12	
Thenardite	Na_2_SO_4_	1	2	2	3	10	
Glauberite	CaNa_2_(SO_4_)_2_					3	
Ca-Langbeinite	K_2_Ca_2_(SO_4_)_3_				1	3	
Yavapaiite	KFe^3+^(SO_4_)_2_	1	1				
Halite	NaCl	24	24	17	2	7	3
Sylvite	KCl	11	12	8	0		1
Belite	2CaO SiO_2_	7	9	8	7		7
Merwinite	Ca_3_Mg(SiO_4_)_2_	6	6	6	8	8	4
Mayenite	Ca_12_Al_14_O_33_		3	3			
Gehlenite	Ca_2_Al [AlSiO_7_]	9	10	10	12	3	9
Feldspar	(Ca,Na,K)(Al,Si)_4_O_8_				4		3
Augite Pyroxene	(Ca,Mg,Fe)Si_2_O_6_				7	10	
Whitlockite	Ca_3_(PO_4_)_2_					10	6
Sum		100	100	100	100	100	100

**Table 6:** Temperature range and percent mass loss for each fly ash and reaction type; temperature range (TR), reaction type (RT), endothermic reaction (Endo), and exothermic reaction (Exo).

TR /°C	A %	RT	B %	RT	C-Fil	RT	TR /°C	C-Boiler %	RT	TR/°C	D %	RT	TR /°C	E %	RT
40–530	1.4	–	1.8	–	1.7	–	45–315	0.4	–	45–350	0.5	–	40–500	0.8	–
– 530–750	– 3.5	– Endo	– 4.7	– Endo	– 3.9	– Endo	315–550	1.1	Endo	– –	– –	– –	– 500–750	– 2.8	– –
							550–810	6.3	Endo						
750–1150	32.8	Endo	34.1	Endo	26.8	Endo	810–1150	5.2	Endo and Exo	350–1150	10.8	Exo	750–1150	5.7	Endo
Sum	37.7		40.6		32.4			13			11.3			9.3	

**Table 7:** Energy content based on DSC signal in N_2_ atmosphere for each fly ash (in kJ/kg).

Fly Ash	A	B	C-filter	C-boiler	D	E
Energy density	94	85	98	394	n.d.	50

**Table 8:** Specific energy content of charging and discharging steps of fly ash C-boiler (in kJ/kg).

Energy Content in kJ/kg	1. Cycle	2. Cycle	3. Cycle
Charging (heating to 880 °C, N_2_ atmosphere)	290	99	105
Discharging (cooling from 880 °C to 350 °C, CO_2_, and H_2_O atmosphere)	73	101	100

**Table 9:** Leaching contents (mg/kg) of heavy metals from ash samples.

Element Components	Limits Value	A	B	C-Filter	C-Boiler	D	E
Al		1.9	2.1	1.51	1.61	3	1471
Sb	0.7 (2.1)	0.05	0.07	0.06	0.05	0.21	0.2
As	2	0.116	0.07	0.13	0.05	-	0.05
Ba	100 (300)	5.61	4.7	5.75	4.8	1.5	74
Pb	10 (30)	294	304	52.55	16.7	24	2.03
Cd	1	0.12	0.21	0.18	0.18	144	0.1
Cr	10 (20)	2.3	2.6	7.8	6.5	1.1	0.9
Co	5	0.1	0.17	0.18	0.18	2.7	0.1
Fe		0.15	0.4	0.24	0.55	0.7	0.12
Cu	50	0.1	0.21	0.2	0.166	1.3	0.18
Mn		0.1	0.17	0.18	0.166	27	0.1
Ni	10	8.9	5.8	1.7	4.8	7.2	1.3
Hg	0.1	0.11	0.18	0.22	0.183	0.01	0.1
Ag	1	0.01	0.01	0.01	0.01	0.6	0.01
Zn	50 (100)	0.07	0.05	0.05	0.05	2.1	0.05
Sn	20	0.1	0.15	0.18	0.18	0.4	0.1
NH_4_	300	0.1	0.17	0.18	0.18	2.2	0.1
Cr(VI)		9.166	5.6	2.7	5.05	0.5	
F	150	1.7	0.6	5.5	5.05	162	0.08
NO_2_–N	15	1	1	1	1	1	1
PO_4_	50	37.5	43.7	33.48	18.3	2.7	5.26
SO_4_		1	1	1	1	147	1
PH		11.9	11.8	12.3	11.1	8.8	11.28
Electrical conductivity (mS/cm)		40.5	43.35	16.71	37.43	31	67
